# Understanding Staffing Disparities in Nursing Homes: Insights from Neighborhood Deprivation

**DOI:** 10.1093/geroni/igaf122.4225

**Published:** 2025-12-31

**Authors:** Jenny Kwon, Xiao Qiu, Samuel Van Vleet

**Affiliations:** University of California, San Francisco, San Francisco, California, United States; Miami University, Miami University, Ohio, United States; Massachusetts General Hospital | Harvard Medical School, Boston, Massachusetts, United States

## Abstract

Low staffing levels in U.S. nursing homes (NHs) remain an ongoing concern for the quality of care. Socioeconomic deprivation in the areas surrounding NH may influence its ability to maintain optimal staffing levels, but this relationship has been understudied. This study was to examine the association between neighborhood deprivation and NH staffing levels. An aggregate dataset of Ohio NHs in 2022 was constructed (N = 610) by merging three public data sources: the Area Deprivation Index (ADI) from the Neighborhood Atlas, Nursing Home Archived Data from the Centers for Medicare & Medicaid Services, and LTCFocus from Brown University. Staffing levels for three types of nursing staff, including registered nurses (RNs), licensed practical nurses (LPNs), and certified nursing assistants (CNAs) were measured by hours per resident day (continuous). Neighborhood socioeconomic disadvantage was assessed using the national ADI. The score was a continuous variable, with a higher score indicating greater deprivation within a neighborhood (range: 0-100). Multiple linear regressions, including control variables, were conducted via SAS. Results show that a higher ADI score is significantly associated with a lower CNA staffing level (p < .05). Negative associations between ADI and RN/LPN staffing levels approach significance (p < 0.1). Given that CNAs provide most hands-on care and closely interact with residents, these findings highlight the need for policy interventions in deprived areas to ensure adequate CNA staffing. Targeted workforce recruitment, improved working conditions, expanded career development and training opportunities, and increased staffing support could help reduce staffing disparities in NHs located in disadvantaged neighborhoods.

